# Supplementary figures and images for: Comparative microfluidic and enzymatic analyses reveal multifaceted snake venom resistance and novel VWF behavior in the opossum Monodelphis domestica
Source: PLoS One. 2025 Sep 19;20(9):e0332686. doi: 10.1371/journal.pone.0332686 (PMC12448354; doi:10.1371/journal.pone.0332686)

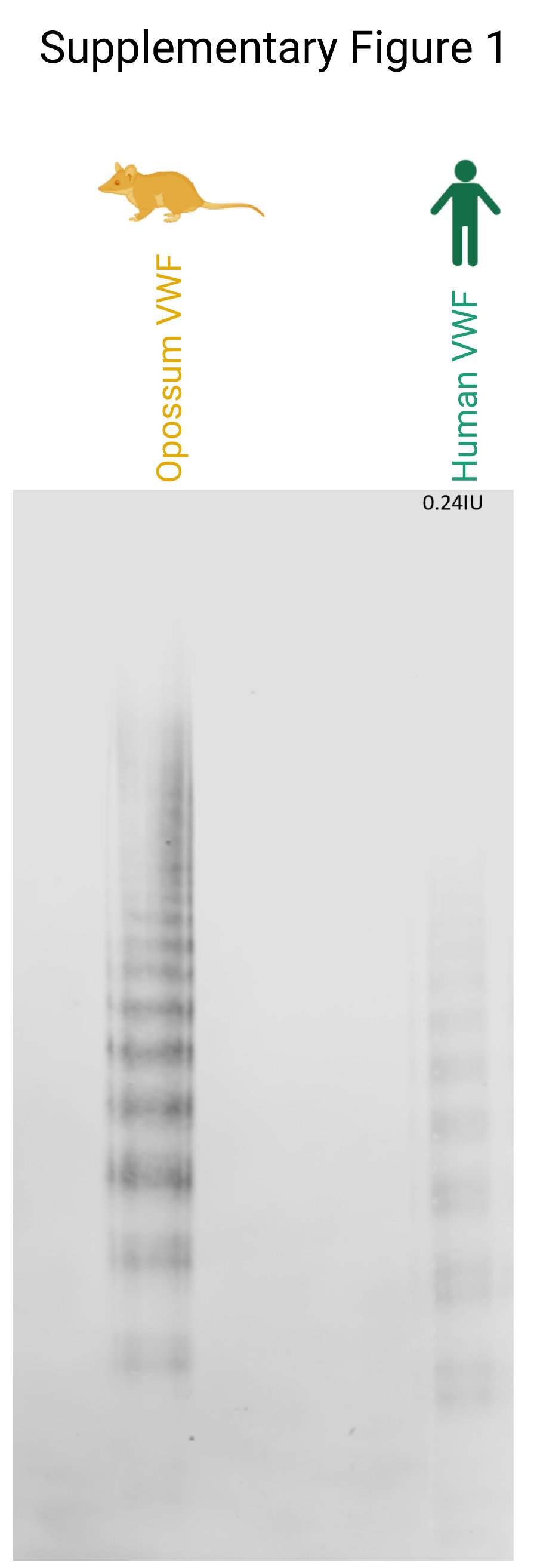

Supplement: S1 Fig — Opossum VWF Multimer Gel 1.6% resolving + 0.8% stacking Tris Glycine LDS-SeaKem HGT Agarose Vertical Multimer Gel (8x8cm vertical). Multimeric structures of von Willebrand factor (VWF) were analyzed using a 1.6% lithium dodecyl sulfate (LDS) agarose gel under non-reducing conditions. Lane 2 contained 30 µL of purified Monodelphis domestica VWF (heated at 60°C for 30 minutes) and 10µl loading buffer. Lane 5 contained 30 µL HumateP (human VWF) as a control (heated at 60°C for 30 minutes) and 10µl loading buffer. The gel was transferred to a nitrocellulose membrane and visualized by fluorescence with anti-human VWF antibody (Dako A0082) using a Li-Cor Odyssey CLx Imaging System (Li-Cor). (TIF) [file pone.0332686.s001.tif]

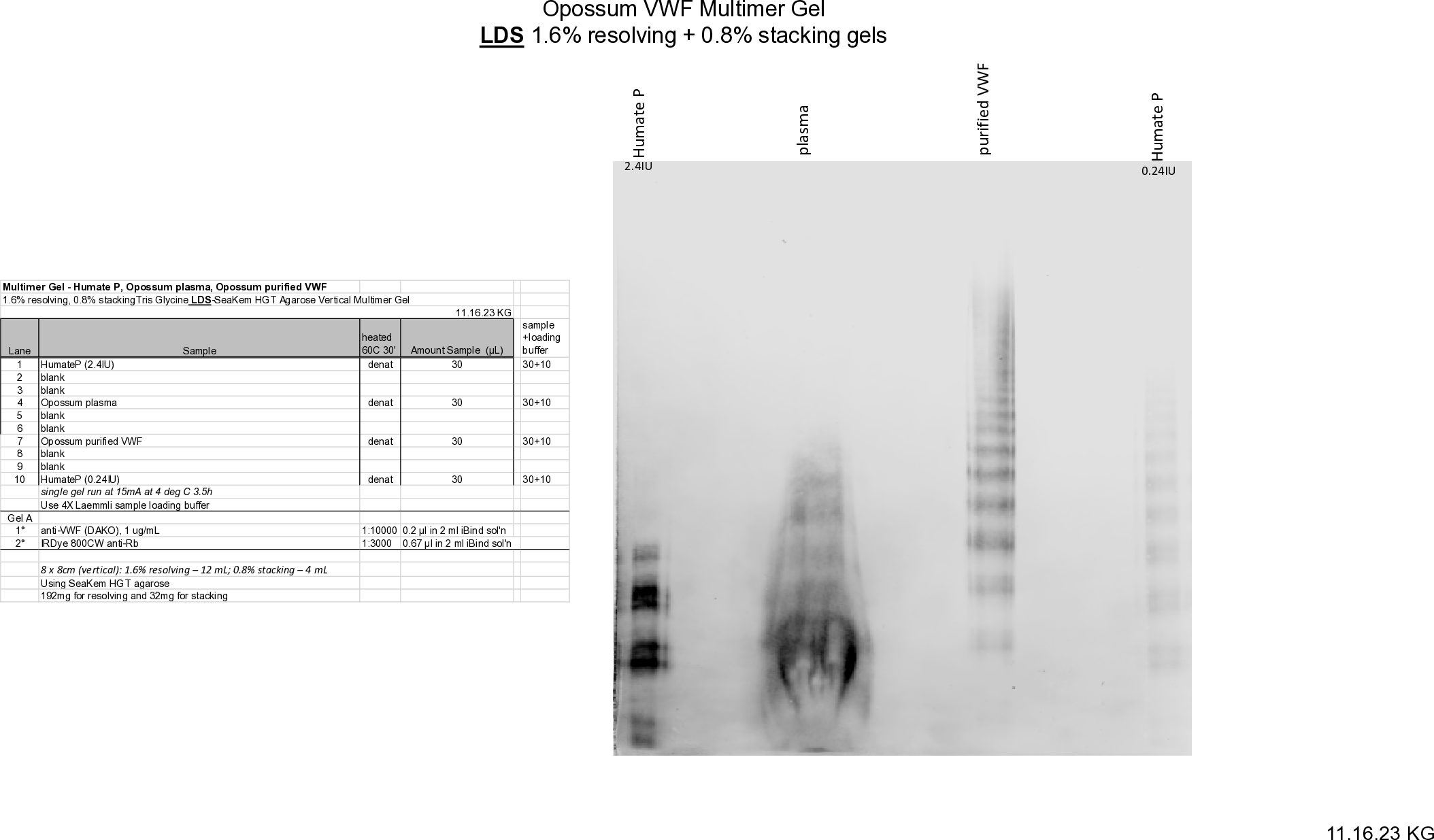

Supplement: S1 Raw Images — Original, unaltered image corresponding to the VWF multimer gel shown in S1 Fig. Includes full gel context and supporting image data used for visualization. (TIF) [file pone.0332686.s002.tif]
