# Supplementary material for: Comparative microfluidic and enzymatic analyses reveal multifaceted snake venom resistance and novel VWF behavior in the opossum Monodelphis domestica
Source: PLoS One. 2025 Sep 19;20(9):e0332686. doi: 10.1371/journal.pone.0332686 (PMC12448354; doi:10.1371/journal.pone.0332686)
Supplement: Attachment — Submitted filename: ResponseLetter_PONE-D-25-27609R1.pdf [file pone.0332686.s004.pdf]

Response to reviews (our responses in bold text):

We thank both reviewers for their thoughtful and constructive feedback on our manuscript. We were pleased to see enthusiasm for the dataset and overall study design, and we have revised the manuscript to improve clarity, strengthen the rationale for experimental progression, and more clearly connect our results to the role of von Willebrand Factor (VWF). In particular, we added new contextual framing throughout the Introduction and Results section, clarified figure references, corrected terminology, and incorporated a dedicated Statistical Analysis section in response to reviewer suggestions. Additionally, we have reworked much of the Discussion section to both shorten its length and explicitly connect the discussed points to the updated framing language in the Introduction and Results. Below, we address each comment in detail, indicating the specific changes made to the text and our rationale where appropriate. We appreciate the reviewers' engagement and believe the manuscript is now stronger and more accessible as a result.

Reviewer #1: In this manuscript, Matthew Holding and colleagues investigated the venom resistance of M. domestica. In different experiments, they examined whether the decreased binding between VWF and botrocetin results in functional resistance to coagulopathic effects, and whether M. domestica is capable of neutralizing venom components beyond CTLs.

I have several questions and remarks on the current version of this manuscript:
-Overall, it is nice data but the focus on VWF (as was expected from the title and abstract) is quickly lost, going to general resistance to coagulopathic effects. Therefore, I would suggest to change the title.

We appreciate the need for clarity of message in our title. The inclusion of VWF in the title emphasizes the key role of VWF in platelet and fibrin deposition as the known major target of botrocetin, as well as the novel finding of the behavior of opossum VWF under shear. However, we see the reviewer's point that the prior title placed too much connection between the nature of the resistance assays and VWF specifically. We have changed the title to the following: "*Comparative microfluidic and enzymatic analyses reveal multifaceted snake venom resistance and novel VWF behavior in the opossum Monodelphis domestica*"

While VWF plays a central mechanistic role in venom resistance, we intentionally investigated downstream physiological responses, including resistance to SVMPs, to demonstrate the integrated and systemic nature of the phenotype and to place investigation of species-specific adaptive venom resistance within the same methodological context used in most other studies of resistance to viper venoms (that of inhibiting hemorrhagic SVMPs). This broader systems-level framing aligns with our title and is essential for establishing organism-level functional resistance. We address this

integration of vWF mediated resistance with multifaceted resistance in the introduction on lines 103-105:
"*These findings suggest that small-bodied species share venom SNACLEC resistance, but it remains unclear whether this reflects integrated, physiological resistance to snake venom that includes resistance to multiple protein classes.*"

In addition, often more explanation is needed at the start of a results paragraph, to explain why a certain experiment is performed.

We appreciate the reviewer's suggestion and have revised the manuscript in several places to improve the clarity and logical flow of the Introductory material and into the Results section. Specifically, the final paragraph of the introduction explicitly includes hypotheses and associated data-centric predictions. Then, we added brief explanatory sentences at the beginning of each results paragraph to clearly articulate the rationale behind each experimental step. Additionally, we have made changes to the introduction section that make the justification for each aspect more clear and concise, further justify the metalloproteinase activity tests, and differentiate the VWF elongation results from the rest of the manuscript. The Results section changes are incorporated at the following **locations:**

Line 344 – Comparative microfluidic analysis of VWF behavior

Addition: ***To determine whether evolved sites in M. domestica VWF [as described in 10] result in functional resistance under physiologically relevant conditions, we first assessed VWF elongation, platelet adhesion, and fibrin formation using microfluidic assays that simulate in vivo shear forces***

Line 360 – Prior to platelet adhesion results
Addition: ***We next compared the platelet adhesion response of opossum and human PRP to purified venom SNACLECs to evaluate whether resistance is preserved in integrated clotting dynamics under flow***

Line 415 – Prior to fibrin kinetics paragraph
Addition: ***Due to the complex nature of blood coagulation, we quantified not only the effects of SNACLECs on platelet adhesion but also fibrin deposition so as to evaluate downstream coagulopathic disruptions.***

Line 441 – Whole Blood Aggregation
Addition: ***To further assess species-specific resistance to venom in the milieu of whole blood, we used whole blood aggregometry to test the platelet aggregation response of M. domestica to both whole venom and purified SNACLECs.***

Line 508 – Venom Metalloproteinase Inhibition
Addition: ***To evaluate whether resistance in M. domestica extends beyond VWF-targeting SNACLECs and to test for ecological specificity of serum-based SVMP inhibition, we***

conducted a complete reciprocal cross of 3 viperid species venoms with 3 mammal serums. Venoms were selected to represent 1) a sympatric species (B. jararaca) for which ecological and other data predict opossum resistance, 2) an allopatric species (C. oreganus) for which another mammal (ground squirrels) have evolved venom resistance 3) an allopatric distantly related viper (B. arietans) (see Fig 4 top and middle panels.)

For figure 1 and 2: “As expected, … platelet accumulation on the microfluidic surface was strongly inhibited by botrocetin and convulxin” (L135-136) Here, a short text is needed to explain why this should be expected. This is provided in the material and methods section so it could be removed there from both ‘Microfluidic Assays’ and ‘Aggregation Assays’ paragraphs, and be added to results section.

We thank the reviewer for this helpful suggestion. We have revised the opening sentence of this paragraph in the Results section (now line 360-365) to include a brief explanation of why botrocetin and convulxin were expected to inhibit platelet adhesion in human blood. Specifically, we now state that this is based on prior work demonstrating the strong disruptive effect of these CTLs on platelet function. This clarifies the basis of the expectation for readers unfamiliar with the specific toxins. We also streamlined redundant text in the Methods to avoid repetition.
Updated Text (Line 360-361):
“Control experiments with human PRP showed that platelet accumulation on the microfluidic surface was strongly inhibited by botrocetin and convulxin, and weakly inhibited by aspercetin (Fig 2A-D), which is consistent with prior work demonstrating that botrocetin and convulxin strongly activate or disrupt platelet function in human blood [37,38].”

For figure 4, it is now not clear how this figure adds to the story (especially not with the current title/abstract), as the main research question here focusses on VWF, but in Figure 4 suddenly metalloproteinase inhibition is tested. Also, suddenly different venoms are used compared to Figure 3. The rationale for this should be provided in the results section.

We agree that additional context was needed. We have added specific introduction section material that both justifies and prepares the reader to encounter SVMP assays:

Lines 92-95: ***“This mechanism is further exacerbated by snake venom metalloproteinases (SVMPs), that cleave plasma proteins and break down vascular integrity to release other venom compoents like SNACLECs into circulation, further compounding coagulopathic damage [19,20].”***

Lines 138-140: ***“We extend these tests of species specificity through assays of serum inhibition of the SVMPs to investigate the potential for adaptive, integrated resistance to multiple venom protein classes.”***

We also added a transitional paragraph to the beginning of the Results subsection presenting Figure 4 (line 508-514) to explain how this assay complements the SNACLEC/VWF-focused experiments. Specifically, we clarify that metalloproteinase inhibition was tested to determine whether Monodelphis domestica exhibits broader resistance to venom effects beyond VWF-SNACLEC interactions. We also clarify the ecological rationale for the venom selection, which includes sympatric, partially sympatric, and allopatric species to assess the specificity of serum-based inhibition.

"To evaluate whether resistance in M. domestica extends beyond VWF-targeting SNACLECs and to test for ecological specificity of serum-based SVMP inhibition, we conducted a complete reciprocal cross of 3 viperid species venoms with 3 mammal serums. Venoms were selected to represent 1) a sympatric species (B. jararaca) for which ecological and other data predict opossum resistance, 2) an allopatric species (C. oreganus) for which another mammal (ground squirrels) have evolved venom resistance 3) an allopatric distantly related viper (B. arietans) (see Fig 4 top and middle panels.)"

-Figure 1: the authors conclude that opossum VWF requires a greater shear force to elongate. Is this significant? This is not indicated in the graph.

Thank you for this important request. The difference between the percent elongation of human and opossum VWF is significant at 40 and 60 dyn/cm^2 with p values of 0.021 and 0.027, respectively. We have edited Figure 1 by adding asterisks to denote statistical significance. Additionally, the text and Figure 1 caption have been updated to reflect this information.

In Figure 1 caption: "Asterisks denote statistically significant differences in percent elongation (-value <0.05."

In the text: "Compared to human VWF, opossum VWF required an additional 20 dvn/cm^2 to fully elongate, and demonstrated statistically reduced elongation at shear stresses of 40 and 60 dyn/cm^2." (lines 354-356)

-Supplemental figure 1: from this figure, I'm not convinced with the conclusion that 'there were no obvious differences in multimer distribution'. First, quantification (% to total) of high, intermediate and low molecular weight multimers is needed. Second, an equal concentration should be loaded on the gel to be able to compare, which seems not to be the case here.

We appreciate the reviewer's attention to detail and agree that quantitative comparison of multimer distribution should include both densitometric analysis and equal loading. We have moderated the language here to reflect both the contents of the gel image and our original goal in conducting this multimer analysis, that of confirming the existence of

high molecular weight multimers in this distantly related mammalian species (lines 349-356).

-In Figure 2, images of fibrin formation under flow would be of added value.

Thank you, and we agree. We have edited Figure 2 to display both platelet and fibrin fluorescence. Additionally, Supplementary Video 1 has been added so that the kinetics of platelet adhesion and fibrin generation under flow can be better appreciated.

The human control is lacking in Figure 3, while it was nicely added in Figure 1 and 2. Regarding the lack of this comparison, the conclusion on L277-279 (Aspercetin appears to be able to function nearly as well in opossums as it does in humans in microfluidics assays (in both cases it has weak function), but not in whole blood assays) can't be drawn as aspercetin was not tested here in whole blood assays using human whole blood.

We appreciate the reviewer's concern. Due to the limited availability of purified CTLs such as aspercetin, convulxin, and botrocetin—some of which are rare, previously fractionated venoms—our study prioritized using these reagents in assays that would generate novel comparative data, specifically in Monodelphis domestica whole blood. Human responses to all three CTLs in PRP and whole blood have been extensively characterized in prior studies (e.g., Drabeck et al. 2020; Rucavado et al. 2001), including using the same purified fractions employed here. To address this comment, we have revised the manuscript text to remove new inferences about human whole blood responses and now cite the relevant prior literature more clearly.

Revised text:

Line 480-484: "Due to limited quantities of these purified SNACLECs, we prioritized their use in Monodelphis domestica whole blood. Human platelet responses to these same protein fractions have been previously characterized in PRP [9,17], and we refer readers to those studies for comparative context."

Line 595-596: "Aspercetin appears to be able to function nearly as well in opossums as it does in humans under flow (in both cases it has weak function), but not in whole blood or previous PRP assays [10], highlighting the importance of integrated assays that incorporate the complex milieu of the blood coagulation system as well the dynamic shear forces that act upon [17,39,46]."

In the graphical abstract, aspercetin is mentioned as 'not resistant' in platelet adhesion, while convulxin and botrocetin are mentioned as 'moderately resistant'. This is not correct when looking at Figure 2J-K?

We thank the reviewer for their careful attention. As shown in Figure 2K, opossum

platelet adhesion is statistically significantly preserved in the presence of botrocetin relative to human PRP, reflecting resistance. In contrast, the response to aspercetin in opossum PRP is nearly identical to that in human PRP—indicating a lack of resistance. Convulxin, while severely impairing adhesion in both species, allowed a modest degree of residual adhesion in opossum PRP which we were classifying as resistance. However, we have appropriately changed this interpretation to "Not Resistant" based on the significant thresholds.

Minor:
-The abbreviations CTL and SVMP are used in the introduction, however not written out fully when used for the first time. Please adjust this.

We have checked the manuscript throughout to ensure initial definition (lines 84 and 93) and proper continued usage of these abbreviations. Additionally, following conversations with a colleague that studies these proteins, we have switched to the abbreviation SNACLEC for the venom c-type lectin-like proteins, as this is the more common usage in the field for differentiation botrocetin and related proteins from the true venom CTLs, which are located on a different chromosome.

"Concordant with the whole blood aggregometry analyses" (L145< L150) is mentioned in the results section of Figure 2. However, only microfluidic experiments using PRP are described thus far, and whole blood aggregometry experiments follow in Figure 3. This text should be adjusted.

We agree with the reviewer and appreciate the clarification. To avoid referencing data that the reader has not yet encountered, we have revised the section to remove comparison with results that are not yet presented:
Lines 373-381: ***"The effect of botrocetin on platelet adherence to the microchannel was decreased in M. domestica (Fig 2 B, F, J): botrocetin caused a more severe defect in the total extent of platelet adhesion than aspercetin in human PRP, but a less severe defect than aspercetin in opossum PRP (Fig 2I-K). Aspercetin and convulxin caused a similar response in both species (Fig 2K). The effect of botrocetin on platelet adherence to the microchannel was also significantly decreased in M. domestica when compared to the same in human (Fig 2 B & F, K). In human PRP, botrocetin caused a 66 ± 16% reduction in the overall extent of platelet adhesion compared to the negative control, whereas it only caused a 39 ± 15% reduction in opossum PRP (Fig 2K"***

-In the results section, refer to the specific subfigures (e.g. Figure 3B instead of Figure 3).

We have updated all relevant figure references in the Results section to indicate specific subpanels (e.g., Figure 3B, 3C) rather than general references (e.g., Figure 3), to improve clarity and guide the reader more precisely to the supporting data.

-Typo in the graphical abstract: sheer stress should be shear stress. Also, the shear stress mentioned here is 80 dyn/cm2 while 60 dyn/cm2 is mentioned in the figure legends.

"sheer" has been corrected to "shear." Regarding the shear stress values, the reviewer is correct that the main experiments (Figures 2 and 3) were conducted at 60 dyn/cm², while Figure 1 specifically tested a range of shear rates to determine the threshold at which VWF fully elongates. As described in the Figure 1 legend, opossum VWF required 80 dyn/cm² for full elongation, compared to 60 dyn/cm² for human VWF. The graphical abstract reflects this specific result from Figure 1.

Reviewer #2:

This article discusses the resistance to envenomation of a small and poorly studied species of Opossum, focusing on coagulopathic effects. The article provides robust information by covering different types of coagulopathic activities to better understand the effects of resistance to envenomation, as well as using different species and isolated toxins, considering the animal ecology and its possible targets of resistance to envenomation. Therefore, I recommend the publication of the article, which brings new information about prey-predator coevolution (Arms race), contributing scientifically to a better understanding of the phenomenon.

Minor suggestions:
Please include an item in the Methodology section to describe statistical analysis used on the article

We appreciate this helpful suggestion. We have now added a dedicated Statistical Analysis section to the Methods, summarizing the statistical approaches used in our study. This includes details on the normalization of elongation assays (Figure 1), ANOVA testing with Fisher's LSD correction for microfluidic assays (Figure 2), and dose-response curve fitting with a four-parameter logistic model for metalloproteinase inhibition assays (Figure 4). This section can be found on line 323:
"Statistical analysis
For elongation assays under varying shear stress conditions, percentage elongation was normalized relative to the 0 dyn/cm² condition (set as 0% elongation) and the 200 dyn/cm² condition (set as 100% elongation) for each species. Data are presented as mean ± standard deviation (SD) based on n = 10 replicates per condition. Significance was assessed using Welch's unpaired t-test.
For comparisons of platelet adhesion and fibrin lag time, statistical significance was assessed using one-way ANOVA with Fisher's Least Significant Difference (LSD) post-hoc correction. Significance thresholds were defined as follows: p ≤ 0.05 (*), p ≤ 0.01

(**), p ≤ 0.001 (***), and p ≤ 0.0001 (****). Data are presented as mean ± standard deviation (SD). Platelet adhesion values were normalized to the control condition for each individual sample to account for variation in platelet counts across donors or animals. Fibrin fluorescence intensity was normalized to background fluorescence (0) and maximum fluorescence (1).
For SVMP inhibition assays dose-response curves were fit using the drc package [36] in R, applying a four-parameter logistic model to serum treatments from Monodelphis domestica, O. beecheyi, and H. Sapiens sera. ED_{90} values were calculated from these fitted curves to quantify the serum volume required to inhibit 90% of venom gelatinase activity."

Line 59 – please include the description of CTLs here – once it´s the first time that the abbreviation is described

We now define this abbreviation at first use, but also have switched to the alternative more phylogenetically-specific designation of SNACLEC rather than CTL.

Line 300 – verify the reference

We have removed this reference.

Suggestion on figure 1: Do the AUC curves to statistically compare between Human and oposum shear stress could enrich the results.

We appreciate the reviewer's suggestion, and we agree that statistical analysis of the data presented in Figure 1 adds value. However, area under the curve (AUC) calculations are arbitrary in the sense that they depend on the range of data being assessed. For example, if we calculate AUC for human and opossum percent elongation for a range of 0-200 dyn/cm^2, the difference is not significant with a p-value of 0.0815. If we instead truncate the data to the range of 0-100 dyn/cm^2, the difference in AUC is significant with a p-value of 0.0179. Therefore, instead of using AUC to assess significance, we used Welch's unpaired t-test to compare human and opossum VWF elongation at each shear tested, and discovered statistically reduced elongation in opossum VWF at shear stresses of 40 and 60 dyn/cm^2. We are grateful that this comment provoked us to explore this more rigorous analysis, as it allowed us to expound in the text:

Lines 354-356: "Compared to human VWF, opossum VWF required an additional 20 dyn/cm^2 to fully elongate, and demonstrated statistically reduced elongation at shear stresses of 40 and 60 dyn/cm^2."
