# Supplementary material for: Comparative microfluidic and enzymatic analyses reveal multifaceted snake venom resistance and novel VWF behavior in the opossum Monodelphis domestica
Source: PLoS One. 2025 Sep 19;20(9):e0332686. doi: 10.1371/journal.pone.0332686 (PMC12448354; doi:10.1371/journal.pone.0332686)
Supplement: Attachment — Submitted filename: Response_revision2.docx [file pone.0332686.s005.docx]

Response to reviewers **(our responses in bold):**

Reviewer #1: The authors have adequately and extensively addressed my comments. I have no further questions or comments.

**We thank the reviewer for their previous comments and for look reviewing our changes.**

Reviewer #2: The authors have satisfactorily addressed the issues raised in the first review. Only a few editing issues should be considered for submission.

**We appreciate the reviewer’s attention to these additional details and have implemented all suggested changes.**

Line 75 - different font from the rest of the text

**Font changed to Roboto**

on line 419, replace the term C-type lectins for SNACLECs.

**SNACLECs added.**

line 455: italic missing on B. jararaca

**“B. jararaca” has been italicized.**

line 557: missing parentesis
**Parentheses added**

references with a different font from the rest of the text

**References font changed to Roboto**

duplicated references ( 49 to 51)

**We have removed the duplicated references.**
